# 16S rRNA gene sequencing of stool samples collected from patients with latent tuberculosis infection before, during, and two months after treatment with 3HP or 4R

**DOI:** 10.1186/s13104-023-06370-7

**Published:** 2023-06-12

**Authors:** Marie Nancy Séraphin, Julia Bellot, Charles A. Peloquin, Volker Mai

**Affiliations:** 1 Emerging Pathogens Institute, University of Florida, Gainesville, FL, 32610 USA; 2 Department of Medicine, Division of Infectious Diseases and Global Medicine, University of Florida, Gainesville, FL USA; 3 Infectious Disease Pharmacokinetics Laboratory, College of Pharmacy, University of Florida, Gainesville, FL USA; 4 Department of Epidemiology, College of Public Health and Health Professions, College of Medicine, University of Florida, Gainesville, FL USA

**Keywords:** Latent tuberculosis infection, Rifamycins, 3HP, 4R, Gut microbiome, Dysbiosis

## Abstract

**Objective:**

We present 16s rRNA gene sequencing (V1-V2 region) and sample data from a pilot observational cohort study to describe the gut microbiota dynamics of subjects with latent tuberculosis infection (LTBI) treated with daily 600 mg rifampicin for four months (4R) or a weekly dose of 900 mg combination of rifapentine and isoniazid for three months (3HP). Our objectives were to (1) document changes to the gut microbiota immediately following exposure to the rifamycins and (2) document recovery to baseline two months after treatment completion.

**Data description:**

We enrolled six subjects with subjects with LTBI and prospectively followed them for 5–6 months. Each subject provided stool samples before, during, and two months after treatment. Six healthy controls were sampled in parallel with the patients with LTBIs. We report amplicon sequence variants (ASVs) and taxonomic assignments for 60 stool samples. Additionally, we provide access to the raw amplicon sequences, and subject responses to questionnaires about their diet, medication, and lifestyle changes over the study follow-up period. Furthermore, we provide the concentration of the parent and partially active rifamycin metabolite concentrations measured validated LC-MS-MS assays of phosphate buffer washes of the stool samples collected from the LTBI participants. This comprehensive dataset is a valuable resource for future systematic reviews and meta-analyses of the impact of LTBI therapy on the gut microbiota.

## Objective

Emerging evidence suggests the gut microbiome could be a major player in the fight against tuberculosis (T.B.) [1, 2]. The extant literature suggests a bidirectional interaction whereby through its nurturing and maturation of the immune system, the healthy gut microbiota can support the host in resisting challenges by respiratory pathogens such as *Mycobacterium tuberculosis* (Mtb*)* [1]. However, alterations to the normal microbiome, or dysbiosis, could potentially lead to a loss of anti-TB immunity [1, 3]. This raises important public health concerns, especially if prophylaxis to prevent TB disease progression could leave treated LTBI patients at increased risk of Mtb reinfection or TB treatment failure [4]. The data presented herein were collected as part of a pilot pre-post cohort study to investigate the extent of gut dysbiosis resulting from exposure to rifamycin during LTBI treatment and document recovery to baseline post-treatment completion. We recruited LTBI patients at two Florida Department of Health (FDOH) TB clinics between February and October 2019 after they had accepted to initiate treatment with a rifamycin-based regimen [5]. We also recruited a healthy control population from the community. Eligible participants were 15–65 years of age and HIV-negative. We excluded from study participation those who reported taking antibiotics a month prior to study enrollment, were diabetic, pregnant or became pregnant during study follow-up, or were breastfeeding, or on regular therapy for any chronic condition. The results of the main study have been submitted and are under review. The raw 16 S rRNA amplicon sequences and sample data presented here may be of interest to researchers working on a systematic review and meta-analysis on the impact of LTBI therapy on the gut microbiota.

## Data description

### LTBI diagnosis and treatment

FDOH clients who tested positive on a tuberculin skin test (TST) or interferon-gamma release assay (IGRA) blood test were diagnosed with LTBI following a chest x-ray and symptoms screening to rule out TB disease [6]. Healthy controls were LTBI negative by self-report of a negative TST or IGRA, and/or were US-born with no history of travel to a TB endemic country. LTBI patients received either four months of daily self-administered rifampicin (4R, n = 4) or three months of weekly rifapentine and isoniazid (3HP, n = 2) by direct observation. The rifamycins (rifampicin and rifapentine) and isoniazid were dosed based on the patients’ weight in kg, for a maximum dose of 900 mg of rifapentine and isoniazid administered weekly, or 600 mg daily rifampicin.

### Stool sample collection

The patients receiving 4R were sampled at time points 0, 1, 2, 4, and 6 months. The patients receiving 3HP were sampled at 0, 1, 2, 3, and 5 months. All control participants provided stool samples at 0, 1, 2, 4, and 6 months intervals. At enrollment, participants were provided a kit that included a stool collection bucket, a 5ml screw top tube containing 3ml of RNAlater stabilization solution and 3 glass beads, wooden stick, and instructions to collect about 1 g of stool at home and ship to our lab in a prepaid bubble mailer. At monthly (30-day window) phone follow-up visits, we collect information on treatment side effects, changes to treatment regimen, and changes to diet from baseline. If LTBI participants reported changes to treatment dosage, or changes to the regimen, we abstracted the data from the medical record.

### DNA extraction and 16 S amplicon sequencing

DNA extraction used a modified Qiagen fecal DNA extraction protocol with an initial mechanical lysis bead-beating step after two phosphate buffer (PBS) wash steps, as previously described [7]. Procedures to profile the gut microbiota via amplification of the V1-V2 hypervariable regions of the bacterial 16 S rRNA were performed as previously described [8]. Equimolar amounts of the amplicon libraries were pooled for paired-end sequencing (250 × 2) using the Illumina Mi-Seq platform (Illumina®, San Diego, CA).

### Parent and metabolite rifamycins concentration measurement

A modification of a validated liquid chromatography-tandem mass spectrometry (LC-MS-MS) assay developed in the University of Florida Infectious Disease Pharmacokinetics Laboratory (IDPL) was used to measure the concentrations of the parent rifampin, rifapentine, and their major metabolite, desacetylrifampicin and 25-O-desacetylrifapentine, respectively in the supernatant recovered during the first PBS wash step of our DNA extraction protocol. The LC-MS-MS assay was performed on a Thermo Acella high-performance liquid chromatography system and a Thermo Ultra triple-quadrupole mass spectrometer with a Dell computer and the Thermo Xcaliburdata management system.

### Gut microbiota profiling

Each participant provided five stool samples for a total of 60 samples included in the study. We generated 6,407,447 16 S sequencing reads with an average of 97,703 ± 20,189 reads per LTBI patient sample and 115,878 ± 31,784 reads per healthy volunteer sample. The sequences were processed and clustered into amplicon sequence variants (ASVs) using DADA2 [9] and assigned to taxonomic class using the naïve Bayesian classifier method [10] and the Ribosomal Database Project (RDP) v16 training set [11]. We applied prevalence filtering to retain phyla if present in at least 5.0% of all samples, and all unclassified reads at the phylum level were filtered out. The 60 raw amplicon sequences were deposited into NCBI under accession number PRJNA772261. The ASVs along with the associated sample data are archived on Figshare (Table 1**)**.

**Table 1:** Overview of data files/data sets

Label	Name of data file/data set	File types (file extension)	Data repository and identifier (DOI or accession number)
*Data file 1*	*Gut microbiome dynamics associated with rifamycin therapy for latent tuberculosis infection*	.*fastq.gz*	NCBI Sequence Read Archive PRJNA772261 [12]
*Data file 2*	*Gut microbiome amplicon sequence variants (ASVs) and sample data from patients with tuberculosis infection before, during, and after treatment with rifamycins*	.*rds*	FigShare 10.6084/m9.figshare.21381930[13]

## Limitations

The main limitation of the data is the sample size, which fell short of the planned enrollment of 20 LTBI participants. The number of eligible LTBI patients who accepted to be treated was small and many refuse study participation because they were uncomfortable with the stool collection protocol.

One of our objectives was to document the extend to reach the gut microbiota recovered to baseline state, following completion of LTBI therapy. We assessed gut microbiota in a single stool sample collected two months after treatment was completed. During the follow-up period, some participants reported taking antibiotic to treat other conditions.

For most of our participants, the post-treatment follow period coincided with the Thanksgiving and Christmas holidays and the first few weeks of stay-at-home orders to control COVID-19 spread. Some participants reported changes in their diet as well as drinking alcohol more heavily during these times. As this was an observational study, participants were not given any specific diet, lifestyle, or behavioral change directives. Although, LTBI participants were advised by the TB clinic not to consume alcohol during therapy to prevent liver injury.

## Data Availability

The data described in this Data Note can be freely and openly accessed on FigShare under 10.6084/m9.figshare.21381930 [13]. The raw amplicon sequences can be freely and openly accessed on NCBI under accession number PRJNA772261 [12]. Please see Table 1 for details and links to the data.

## Abbreviations

3HP: Three months of weekly isoniazid in combination with rifapentine
4R: Four months of daily rifampin
IGRA: Interferon gamma release assay
LTBI: Latent tuberculosis infection
rRNA: Ribosomal ribonucleic acid
TB: Tuberculosis
TST: Tuberculin skin test
TBI: Tuberculosis infection
